# Semantic tagging of and semantic enhancements to systematics papers: ZooKeys working examples

**DOI:** 10.3897/zookeys.50.538

**Published:** 2010-06-30

**Authors:** Lyubomir Penev, Donat Agosti, Teodor Georgiev, Terry Catapano, Jeremy Miller, Vladimir Blagoderov, David Roberts, Vincent S. Smith, Irina Brake, Simon Ryrcroft, Ben Scott, Norman F. Johnson, Robert A. Morris, Guido Sautter, Vishwas Chavan, Tim Robertson, David Remsen, Pavel Stoev, Cynthia Parr, Sandra Knapp, W. John Kress, Chris F. Thompson, Terry Erwin

**Affiliations:** 1 Bulgarian Academy of Sciences & Pensoft Publishers, 13a Geo Milev Str., Sofia, Bulgaria; 2 Plazi, Zinggstrasse 16, Bern, Switzerland; 3 Pensoft Publishers, 13a Geo Milev Str., Sofia, Bulgaria; 4 Nationaal Natuurhistorisch Museum Naturalis, Netherlands; 5 The Natural History Museum, Cromwell Road, London, UK; 6 The Ohio State University, Columbus, OH, USA; 7 University of Massachusetts, Boston, USA & Plazi, Zinggstrasse 16, Bern, Switzerland; 8 IPD Bohm, Karlsruhe Institute of Technology, Germany & Plazi, Zinggstrasse 16, Bern, Switzerland; 9 Global Biodiversity Information Facility, Copenhagen, Denmark; 10 National Museum of Natural History, 1 Tsar Osvoboditel blvd., Sofia, Bulgaria; 11 Encyclopedia of Life, Washington, DC, USA; 12 Smithsonian Institution, Washington, DC, USA

**Keywords:** Semantic tagging, semantic enhancements, systematics, taxonomy

## Abstract

The concept of semantic tagging and its potential for semantic enhancements to taxonomic papers is outlined and illustrated by four exemplar papers published in the present issue of ZooKeys. The four papers were created in different ways: (i) written in Microsoft Word and submitted as non-tagged manuscript (doi: 10.3897/zookeys.50.504); (ii) generated from Scratchpads and submitted as XML-tagged manuscripts (doi: 10.3897/zookeys.50.505 and doi: 10.3897/zookeys.50.506); (iii) generated from an author’s database (doi: 10.3897/zookeys.50.485) and submitted as XML-tagged manuscript. XML tagging and semantic enhancements were implemented during the editorial process of ZooKeys using the Pensoft Mark Up Tool (PMT), specially designed for this purpose. The XML schema used was TaxPub, an extension to the Document Type Definitions (DTD) of the US National Library of Medicine Journal Archiving and Interchange Tag Suite (NLM). The following innovative methods of tagging, layout, publishing and disseminating the content were tested and implemented within the ZooKeys editorial workflow: (1) highly automated, fine-grained XML tagging based on TaxPub; (2) final XML output of the paper validated against the NLM DTD for archiving in PubMedCentral; (3) bibliographic metadata embedded in the PDF through XMP (Extensible Metadata Platform); (4) PDF uploaded after publication to the Biodiversity Heritage Library (BHL); (5) taxon treatments supplied through XML to Plazi; (6) semantically enhanced HTML version of the paper encompassing numerous internal and external links and linkouts, such as: (i) vizualisation of main tag elements within the text (e.g., taxon names, taxon treatments, localities, etc.); (ii) internal cross-linking between paper sections, citations, references, tables, and figures; (iii) mapping of localities listed in the whole paper or within separate taxon treatments; (v) taxon names autotagged, dynamically mapped and linked through the Pensoft Taxon Profile (PTP) to large international database services and indexers such as Global Biodiversity Information Facility (GBIF), National Center for Biotechnology Information (NCBI), Barcode of Life (BOLD), Encyclopedia of Life (EOL), ZooBank, Wikipedia, Wikispecies, Wikimedia, and others; (vi) GenBank accession numbers autotagged and linked to NCBI; (vii) external links of taxon names to references in PubMed, Google Scholar, Biodiversity Heritage Library and other sources. With the launching of the working example, ZooKeys becomes the first taxonomic journal to provide a complete XML-based editorial, publication and dissemination workflow implemented as a routine and cost-efficient practice. It is anticipated that XML-based workflow will also soon be implemented in botany through PhytoKeys, a forthcoming partner journal of ZooKeys. The semantic markup and enhancements are expected to greatly extend and accelerate the way taxonomic information is published, disseminated and used.

## Introduction

“Adapt or die” is certainly one of the most well-known fundamental principles of the theory of natural selection. If we want to paraphrase this principle so that it applies to the dynamic and challenging world of academic publishing, it seems that we have to progress from the recently popular “go online or die” to the rapidly emerging “link yourself or die”. Within just the past few years, several important components of the Semantic Web, such as cross-linking, semantic tagging, data publication, data sharing, data aggregation, etc., have become ordinary components in the vocabulary of the biodiversity scientists. Moreover, we have already several prototypes of the “articles of the future” published in the form of exemplar papers (e.g., Pyle et al. 2008, Johnson et al. 2008, Fisher et al. 2008, Shotton et al. 2009, Miller et al. 2009, Sharkey et al. 2009).

The history of semantic enhancements to biodiversity papers is short but dynamic, starting perhaps as far back as the beginning of the present decade, exemplified by the articles of Erwin and Johnson (2000), Page (2006), Shotton (2009) and others. Perhaps the first taxonomic article to show how embedded hyperlinks may bring vital additional information to a published taxonomic text (i.e., to enhance it) is the famous “Chromis article” of Pyle et al. (2008). Shortly after its publication, use of hyperlinks to external resources, such as Zoobank (http://www.zoobank.org), Morphbank (http://www.morphbank.org), Genbank (http://www.genbank.org), and others, started to become, if not ordinary, a relatively unremarkable feature of taxonomic papers (e.g., Miller et al. 2009, Talamas et al. 2009, Mengual and Ghorpadé 2010). The hyperlinking of text strings has often been enriched through additional enhancements, such as publication of datasets (Costello 2009, Smith 2009, Chavan and Ingwersen 2009, Miller et al. 2009, Penev et al. 2009a) and interactive keys (Sharkey et al. 2009, Penev et al. 2009b).

Hyperlinking of text strings within a paper or links to external sources are useful and widely used methods, however they can no longer be considered a “cutting edge” feature of text processing and publishing practices. A completely new world of data mining and processing of taxonomic texts through semantic XML mark up has been recently advanced by the efforts of a group of enthusiasts around Plazi (http://www.plazi.org, see also http://en.wikipedia.org/wiki/Plazi and Agosti and Egloff 2009). Plazi articulated some truly innovative concepts and tools, such as an electronic form of the “taxon treatment” concept (Sautter et al. 2007, Agosti et al. 2007), TaxonX and TaxPub XML schemas for either marking up legacy literature (http://www.taxonx.org, http://sourceforge.net/projects/taxonx), or to serve prospective publishing (http://sourceforge.net/projects/taxpub), respectively. A special software tool, GoldenGATE, was also developed by Plazi (together with IPD Böhm at the Karlsruhe Institute of Technology, Germany) to facilitate the process of marking up of published taxonomic works (http://plazi.org/?q=GoldenGATE). Major efforts in this direction were also invested by the Literature Working Group of TDWG (http://wiki.tdwg.org/Literature) to elaborate the TaXMLit schema as a future TDWG standard (see also (http://www.sil.si.edu/digitalcollections/bca/documentation/taxmlitv1-3intro.pdf).

The rapid development of bioinformatics thanks mostly to the efforts of enthusiastic groups of people and organisations, e.g., the Taxonomic Database Working Group or TDWG (http://www.tdwg.org), the Global Biodiversity Information Facility, or GBIF (http://www.gbif.org), GenBank (http://www.genbank.org), ZooBank (http://www.zoobank.org), Morphbank (http://www.morphbank.org), Encyclopedia of Life, or EOL (http://www.eol.org), Biodiversity Heritage Library, or BHL (http://www.biodiversitylibrary.org), as well as of the so-called “bottom-up” initiatives, such as Wikipedia (http://www.wikipedia.org), Wikispecies (http://www.species.wikimedia.org), Wikimedia (http://www.wikimedia.org) and others has led to some “technological lagging” in applying new technologies by the publishing industry. Publishers have not adapted so quickly to the active developments of bioinformatics tools. Nevertheless, during the last few years, some innovative exemplar papers started to elucidate the essence of the next generation of journal articles in taxonomy. Two of them have greatly inspired the ZooKeys team to pursue new approaches to publication and dissemination and have had a substantial impact on the current paper. These are the “Neglected disease” semantically enhanced exemplar paper by Shotton et al. (2009) and the “Elsevier Grand Challenge” paper by Page (2010) and our model incorporates some elements from these. Other sources of inspiration include some web-based projects and tools, particularly uBio (http://www.ubio.org) and iSpecies (http://www.ispecies.org).

The aim of the present paper is to briefly describe semantic tagging and semantic enhancement concepts and their application to publishing in biological systematics.

It describes the editorial workflow pioneered by ZooKeys to make the process of tagging, linking and proper dissemination of taxonomic texts technologically and economically viable. We also will demonstrate the great advantages that these new methods provide not only to biodiversity publishing efficiencies, but also to better retrieval, use and future re-use of published content.

## Semantic tagging and semantic enhancements in systematics

Semantic tagging is generally considered to be a method of assigning markers, or tags, to text strings to identify their meaning so that the string and its meaning can be made discoverable and readable not only by humans but also by computers. There are several computer languages developed to provide semantic tagging, the most popular of them being the eXtensible Markup Language (XML) (see next section). Special machine-readable XML documents called “XML schemas” constrain the valid use of each tag, and so provide the background for semantic tagging. For example, in basic XML one can tag the name Drosophila melanogaster with the tag TaxonName. Provided users’ tools take care to uniformly use this for an actual taxon name, there will be no semantic discord among or within documents about what is a taxon name, and software tools can easily be built to exploit these implicit community agreements about meaning. Special languages, namely XML-Schema and the XML Document Type Definition (DTD) can express syntactic restrictions on documents that enforce some context on the use of community-designed controlled vocabulary. When documents comply with these restrictions, it is then possible to write and support software to perform meaningful searches within or across documents, to transform documents from one form to another (e.g. from XML to PDF or HTML), or to facilitate a standardised way for archiving and computer retrieval of the whole document.

**Figure 1.**
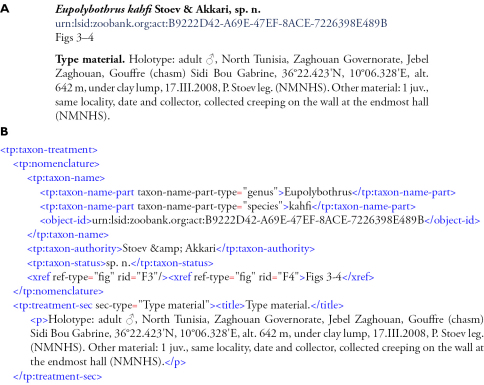
Conventional layout of a standard taxonomic publication in PDF format (A) and the same portion of text in XML-tagged format (B). Explanations: The sign “<” incidates the start tag and the symbol “</” indicates the end tag; the tag <tp:taxon-treatment> denotes the start of the treatment and the tag </tp:taxon-treatment> (not visible here) marks up the end of the treatment within the text of the paper. The tags <tp:treatment-sec> and </tp:treatment-sec> denote the start and end of a particular section of the treatment, in this case the type material data (labelled as <title> Type material.</title>)

At the forefront of informatics research, visions of a fully Semantic Web are advancing (http://en.wikipedia.org/wiki/SemanticWeb) but these seem to remain over the horizon for robust scientific publishing. It is beyond the scope of the present paper to cover in fine detail the vast and extremely dynamic area of semantic tagging, even in the sense we use it. We illustrate how tagging works in taxonomic publications with the following simple example (Fig. 1). Thanks to tagging, computers can recognise portions delimited between the start and end tags to have a certain meaning, thus they can retrieve tagged texts, extract information from them, direct elements to databases and so on.

Semantic tagging is often related to semantic enhancements providing a good basis for the latter. The terms, however, are not identical. Semantic enhancement to scientific texts can be determined as “anything that enhances the meaning of a published journal article, facilitates its automated discovery, enables its linking to semantically related articles, provides access to data within the article in actionable form, or facilitates integration of data between articles” (Shotton et al. 2009).

In the current mature XML technologies, semantic enhancements are typically used for a better visualization and utilization of published text through various hyperlinks, either within the text or to external resources, while tagging is mostly used to transform a text into a computer-readable form. Tagged text could be presented in a simple, “non-enhanced” form, and vice versa, semantically enhanced papers need not necessarily be based on XML-tagged text. Important new and rapidly developing areas of semantic enhancements include the so-called “mashup” and “linkout” technologies created to utilize data from different online resources (e.g., mapping geographical localities of a taxon harvested from different articles, datasets and websites. Linkout software tools locate strings or identifiers within certain Web resources (e.g., through a taxon name or its persistent identifier), receive back the information (often in XML or JavaScript Object Notation [JSON] formats) and represent a summary of that information on a resulting webpage. Harvesting web resources with the help of so-called “scraper” or “harvester” software can be made dynamically, that is in real time (mostly through APIs, Application Programming Interfaces, when these are available on the source website) or by search/provide functions. 

## The “taxon treatment” concept, TaxonX and TaxPub

The concept of “taxon treatment” is exploited by the Plazi team to model taxonomic publications and explore how much of the text tagging can be done by machine either before or after publication. Following taxonomic paper publishing traditions, an initial definition for the electronic form (Sautter et al. 2007), a taxon treatment can include a formal description of a taxon including sections on nomenclature, morphological characteristics, behavior, ecology, distribution, and specimens examined.

The launch of the electronic taxon treatment concept played a key role in the development of taxonomic tagging methodology. Moreover, it is expected that its influence will increase in the near future. Thus, we consider it necessary to describe the concept here in more detail. 

From the text-processing perspective, a taxon treatment is any “block of text” containing information on a given taxon, that can be delimited from other taxon treatments within the same document by specifying the treatment’s start and end tags. From the viewpoint of the publishing tradition in systematics, the treatment is a block of information on a given taxon that may include some elements of the following: 

1. New taxon description

2. Change of a nomenclatorial status of a taxon (a nomenclatural act)

3. Summary of all previous knowledge on a taxon from literature sources, usually structured in logical pieces, e.g., nomenclature, morphological description, distribution, ecology, biology

4. Summary of all previous knowledge plus newly published data on the same taxon, e.g., localities, ecological/biological observations

5. Summary of newly published data on an already known taxon

6. Summary of treatments of subordinated taxa, for instance a revision or catalog of a genus listing treatments of ALL or SOME of its species is a treatment of that genus

7. Listing of subordinated taxa, e.g., a checklist of a family from a region forms a treatment of that family.

Taxon treatments usually have the form of published conventional texts that could be enhanced by a wide array of tags and external links. More importantly, taxon treatments may be archived, searched, harvested, or linked as separate pieces of information directly related to their respective taxa.

A publication may consist of one or many treatments of different taxa of different taxonomic ranks. One taxon may have more than one treatment within a publication, although the tradition of systematics publishing usually assumes one “core” treatment per taxon within a document.

Taxon profiles generated “on the fly” or extracted through web “scrapers” have several features of treatments (e.g., EOL, NCBI, Wikipedia, or ispecies.org taxon profiles). To be called treatments, however, they have to be published in a static and citable form. It seems necessary to distinguish these two types of taxon profiles (published and dynamic, generated on the fly), although the border between them may sometimes seem vague. The essential feature of a treatment is that it encompasses information published in accordance with both present-day publishing standards and the requirements of nomenclatural codes.

What is not a taxon treatment?

1. A citation of a taxon name within a text, although such a citation usually holds information linked to the particular taxon. For instance, listing of a species within a “plain” checklist cannot be a treatment of that species; a sentence within a text paragraph stating that “taxon X is parasitic on taxon Y” is neither a treatment of taxon X nor of taxon Y

2. A key, because in some cases keys are constructed for related taxa that do not form a taxon (they may form a “species-group” or “taxa-group”, but this is not a taxon unless a name is given to that group). Identification keys, even they are exhaustive for a named taxon, are usually tagged separately from taxon treatments.

3. A single picture or group of pictures of a taxon

4. A single map or group of maps of a taxon

5. Gene sequence(s) of a taxon

6. SDD (Structured Descriptive Data) (or any) matrices, or raw data, or databases. Treatments can be relatively easily generated from databases, however, information on a taxon becomes a treatment when (a) it is published, and (b) corresponds to the aforementioned definition of taxon treatment. 

The TaxonX schema and the TaxPub DTD largely follow the above restrictions which arise from a community of practice rooted in paper publishing. In the electronic era, broader notions of a treatment can easily be added to the electronic forms by simple extension of the schema or DTD, in ways that do not make useless publications with the narrower form. 

Why are taxonomic treatments important? What role do they play in various disciplines? Taxonomic treatments are important because they allow “atomising” taxonomic texts, that is they permit labelling and delimiting a piece of information (e.g., a block of text) linked to a taxon within a document from other similar pieces of information, linked to other taxa. Taxonomic treatments allow a rapid transition from conventional, article-level publishing in the biodiversity science, to treatment-level (or content- or data-level) taxonomic publishing. XML encoded taxonomic treatments facilitate future use, re-use and collation (harvesting and indexing, mashups, linkouts) of data, because computers can recognise data elements within treatments and relate such data to taxon names.

Taxonomic treatments are important because they allow mobilization, retrieval and re-use of any and all taxonomic data published not only in the present day, but also in historical taxonomic literature. Recent and historical treatments can be interlinked through taxon names.

Finally, treatments are important because in a straightforward way they relate information on organisms to the oldest and most widely used identifiers in the history of biology – the taxonomic names of organisms. Through names, and especially through the recently developed global index of taxon names (Global Names Architecture, or GNA, Global Names Index, or GNI, Global Names Usage Bank, or GNUB, see http://www.globalnames.org and http://www.gbif.org) treatments may be linked to any other information in any other branch of science that uses taxonomic names.

To facilitate “atomizing” of taxonomic texts into retrievable and machine-readable forms, we need a computer language and sets of rules and protocols in taxonomic publishing, such as XML (see above for more details). TaxonX is a light markup XML schema developed to encode historical, or legacy, taxonomic literature. It is therefore robust enough to retrieve a great variety of styles used in such literature. TaxPub was developed as an extension of the general Document Type Definitions (DTD) format of the National Library of Medicine of the US (NLM, http://dtd.nlm.nih.gov) to facilitate markup of prospective taxonomic publishing.

The ZooKeys working examples (Stoev et al. 2010, Blagoderov et al. 2010b, Brake and von Tschirnhaus 2010, Taekul et al. 2010) are entirely based on revision #123 available from the SVN trunk of TaxPub (http://sourceforge.net/projects/taxpub). In fact, the present exemplar papers are the first published TaxPub articles in biodiversity science, intended to demonstrate the advantages of the XML-based markup and editorial workflow in the way biodiversity information is being published and disseminated.

## Implementation of tagging and external linking in the editorial process

**Figure 2.**
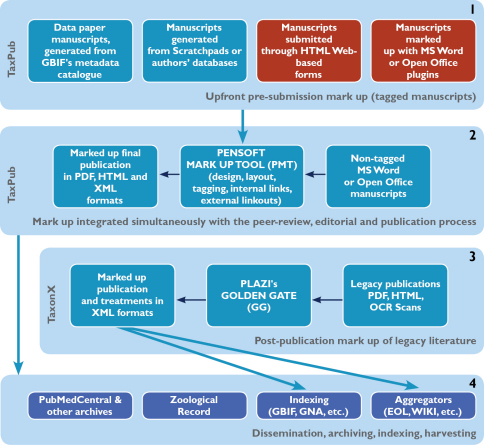
Four stages of an XML-based editorial, publication and dissemination workflow applied in ZooKeys (stages 1, 2, 4) and/or Plazi (stages 3, 4). Forms in blue are either implemented or prototyped, forms in red are in a process of development.

The overall workflow of implementation of tagging of taxonomic texts, either published in legacy literature or within a prospective, XML-based editorial process, is shown in Fig. 2. Tagging of taxonomic text is a quite laborious task, mostly because of the specificity of the domain, e.g., the great variety in use of publishing styles, taxon names (synonymy, homonymy, spelling errors, different concepts for a particular taxon name, etc.), listings of localities (long lists of terms describing a particular locality or collecting event), etc. In most cases, this is being done manually or semi-manually, which may explain why finer granularity mark up has not been used by taxonomic journals thus far. There are two possible ways to solve this problem and optimize the mark up process so that it becomes economically viable.

A straightforward way is to have manuscripts tagged before submission through (i) exports from databases, such as Scratchpads (http://www.scratchpads.eu), GBIF or authors’ personal/institutional databases, or by using (ii) HTML submission forms, or through (iii) TaxPub or other XML Schema based plugins of MS Word or Open Office text processors. The latter method will help authors to write extensive manuscripts of a more complicated structure than those generated from databases or submitted through HTML forms. None of these methods is widely used, to say the least, and (ii) and (iii) simply do not exist yet. There is no doubt, however, that we can anticipate a quick transformation to “automated” generation and submission of manuscripts within the coming years, and surely within the lifespan of the present-day generations of active taxonomists.

The second route to the same output is for publishers to find a way to apply XML tagging within their editorial workflows. As far as it concerns the general article structure, such as title, authors, abstract, introduction, etc., this is not a problem and most major publishers do it. However, once we decide to go to a finer mark up, that is to tag taxon names, taxon treatments, sections within a taxon treatments (nomenclature, morphological description, distribution, type material, examined material with data on localities and specimens, etc.), the difficulties appear hardly surmountable and there is no current working solution for them in biodiversity science, to the best of our knowledge. 

The exemplar papers published in the present issue demonstrate three different approaches to manuscript preparation and submission, ending at the same time in unified semantically enhanced outputs in a form of HTML papers, their XML files intended for computer retrieval and archiving in PubMedCentral, as well as in standard PDF (and print) formats. The paper by Stoev et al. (2010) was submitted as an ordinary Microsoft Word file and all the process of semantic tagging and enhancements were performed in ZooKeys’ Editorial office using the Pensoft Mark Up tool (PMT). The papers of Blagoderov et al. (2010b) and Brake and von Tschirnhaus (2010) were generated and submitted as XML-tagged files by the Scratchpads websites (http://www.sciaroidea.info and http://milichiidae.info); the pre-submisison XML tagging facilitated text processing, which was revised using the same PMT software to create a fully laid out and linked out HTML paper (see also Blagoderov et al. (2010a) for description of the process). Similarly, the paper of Taekul et al. (2010) was submitted as XML-tagged file, generated from the Proctotrupoidea web-based database (http://www.vsyslab.osu.edu).

**Figure 3.**
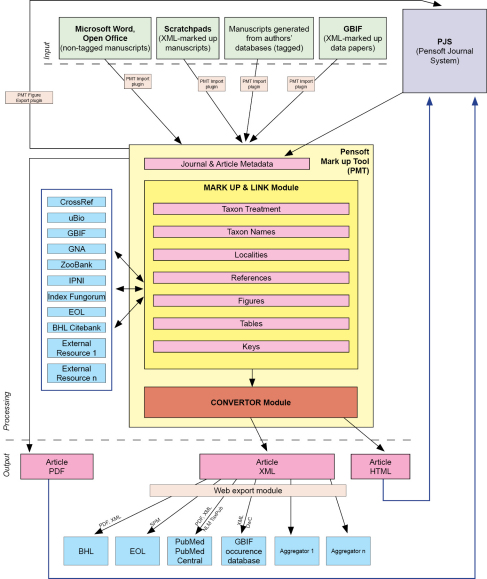
Flowchart of an integrated, XML-based editorial, publishing and dissemination process applied in ZooKeys through the Pensoft Mark Up Tool (PMT).

To implement the two aforementioned routes for XML mark up in prospective taxonomic publishing, we have designed and developed the Pensoft Mark Up Tool (PMT) (Fig. 3). The tool provides the following operations:

1. Importation and retrieval of XML, HTML and InDesign files

2. Interlinking options between PMT and InDesign allowing simultaneous mark up and editorial work 

3. Tagging and autotagging at different granularity levels, according to TaxPub or any other XML schema designed for such purpose

4. Cross-linking of citations within the text and reference list

5. Cross-linking of citations of figures and tables in the text

6. Finding and linking taxon names through http://www.uBio.org and PMT’s own web harvester

7. Providing links to various external sources

8. Exporting the text to a semantically enhanced HTML version of the paper, vizualizing some of the important tag elements, as well as the literature references cited in the text and external links to them (when available)

9. Mapping localities listed in the paper or within separate taxon treatments

10. Generating the Taxon Pensoft Profile page for each taxon name cited in a paper, providing the reader with a quick and up-to-date summary of information on a taxon from certified external sources

11. Offering a possibility to the reader to create their own taxon profiles for taxa of interest

12. Export to a TaxPub XML file, validated for archiving in PubMedCentral and indexing in PubMed

13. XML export of new species descriptions to Encyclopedia of Life, using elements drawn from Dublin Core, TDWG Darwin Core, and TDWG Species Profile Model schemas

14. XML export of treatments or any other tagged information in various formats acceptable by aggregators and indexers, Plazi taken as an example.

**Figure 4.**
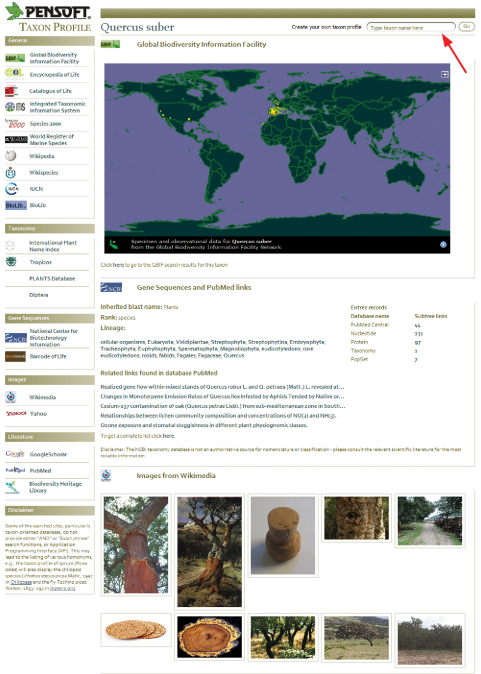
Pensoft Taxon Profile created dynamically by PMT and available through a link to any taxon name mentioned within a paper. In this case, this is the oak species Quercus suber L., cited in a zoological paper (Stoev et al. 2010). The red arrow indicates the “Create your own taxon profile” option, that may be used by the reader to create profiles of any taxon name or to improve search results for taxonomic names cited in the paper.

A special feature of the PMT is to dynamically harvest selected web resources and present the information linked to a certain taxon name on a separate webpage called the Pensoft Taxon Profile (PTP). The PTP module uses uBio (http://www.uBio.org) as a source of taxon names and links them to either the uBio-harvested web resources or through PMT’s own web harvester. The PMT creates profiles of any taxon name mentioned in a paper, independent of its rank or nomenclatural status. An example of a PTP page created for an oak species, Quercus suber, cited in a zoological paper (Stoev et al. 2010) is shown in Fig. 4. This aggregation into taxon pages is similar to that of other projects such as EOL, Scratchpads, iSpecies, BioLib, and iNaturalist.org.

Two classes of selected websites are targeted by the PTP: (1) pillars of biodiversity informatics online (e.g., GBIF, NCBI, EOL, Barcode of Life, Wikipedia, BHL, and others) have dedicated windows showing results for a particular taxon name, or reporting that no results were found (because the lack of results from key online resources could itself be an important finding), and (2) taxon-oriented websites, from which results are displayed only if a particular taxon name was found (e.g., ZooBank, International Plant Name Index, diptera.org and others). 

While new information on the Earth’s biodiversity is added to the World Wide Web every day, many species are still not well represented online. In such cases, the PTP offers the option to “Create your own taxon profile,” which allows users to add, organize, and correct information for particular species (Fig. 4, red arrow).

There is certainly room for many more linking options and modules to be added to PMT so that it makes the process of taxonomic publishing and reading a true pleasure. At the same time, applying tools of this kind may solve long-standing problems with taxonomic mark up and make it cost-efficient and widely used. 

## Four different formats of taxonomic papers and their archiving

The exemplar and forum papers are published in four different formats: (1) print, provide archiving on paper in libraries and to comply with the current requirements of the International Code of Zoological Nomenclature (ICZN), (2) PDF to provide an electronic version identical to the printed one from the publisher’s website, to be archived in PubMedCentral, Biodiversity Heritage Library, as well as in other institutional or personal archives; (3) HTML to provide numerous links to external resources and semantic enhancements to published texts to facilitate interactive reading, as well as to be permanently available on the publisher’s website and through its persistent identifier, the doi number; (4) XML based on the TaxPub DTD to provide an archiving document format for PubMedCentral and a machine-readable copy of the contents to facilitate future data mining.

For reference, we recommend to use either the print or PDF version (the latter provided also through a persistent online identifier, the doi number) and the respective disclaimer is displayed in the beginning of the HTML version.

We consider PubMedCentral as the most appropriate place to archive open access e-versions of taxonomic publications because the whole content of a paper is being stored in both XML and PDF versions. In addition, the figures are archived as separate files. Archiving of the PDF version on BHL provides an additional and very useful cross-link to historical literature through taxon names. Naturally, under the open access model, the online versions of a paper can be disseminated and stored in an unpredictable number of institutional or personal archives. 

## Use and dissemination

We are convinced that the Semantic Web will soon bring entirely new models of publishing and dissemination in systematics and biodiversity science in general. Text tagging and semantic enhancements are certainly not provided for the pleasure and convenience of readers only. The properly tagged texts will be easily harvested and indexed by computers and imported into databases without any human intervention. At any point in the world, taxonomists, ecologists, conservationists and any user will be able to pick up quickly and efficiently most essential information about a taxon, or locality, or even a specimen, such as descriptions, images, maps, keys, gene sequences and references. It only remains for us to act to realize our dream that all this information is available through open access with no barriers to anyone to read and use! The goal of ZooKeys for animal systematics, and soon of PhytoKeys for botanical disciplines is to make this dream a reality.
